# The Impact of a 10-Week Military Training Course on Saudi Medical Recruits' Fitness and Physical Activity Levels

**DOI:** 10.7759/cureus.46593

**Published:** 2023-10-06

**Authors:** Eidan Alzahrani, Faisal M Alyazedi

**Affiliations:** 1 Physical Therapy Department, Prince Sultan Military College of Health Sciences, Dammam, SAU

**Keywords:** pt, body fat percentage, body composition, physical activity, fitness

## Abstract

Background

In preparation for military service, new recruits undergo a physical transformation. We aimed to determine the fitness outcomes and self-reported activity levels of Saudi medical recruits after a 10-week initial military training course (IMTC).

Methods

The cohort comprised 104 recruits aged 25-29 years. Anthropometric variables, including height, body mass index (BMI), body weight (BW), percent body fat (%BF), lean body mass (LBM), waist circumference (WC), and waist-to-height ratio (WtHR), were assessed pre-IMTC and post-IMTC. Physical fitness assessments, including a one-minute sit-up test, push-up test, Cooper's 12-minute run/walk test, and relative maximal oxygen consumption (VO_2_ max), were also evaluated. The International Physical Activity Questionnaire (IPAQ) was used to assess self-reported physical activity.

Results

We found a significant decrease in anthropometric variables following the course, including BW (P = 0.01), BMI (P = 0.01), %BF (P = 0.002), LBM (P = 0.01), WC (P = 0.005), and WtHR (P = 0.003). They also showed significant improvements in the push-up test (P = 0.001), one-minute sit-up test (P = 0.001), 12-minute test (P = 0.001), and relative VO_2_ max (P = 0.001). The comparison of pre-IPAQ with post-IPAQ demonstrated a percentage improvement in walking activity (15%-82%) and vigorous physical activity (17%-49%) after joining the IMTC.

Conclusions

These findings demonstrate that Saudi medical recruits who partake in the IMTC can attain significant improvements in their body composition, physical fitness, and physical activity levels.

## Introduction

Prospective soldiers are required to complete an initial military training course (IMTC) before being drafted into the military. This course is designed to identify those who will most efficiently meet the demands of military service and help them maintain this fitness level throughout their service [1]. Regular physical activity is associated with a decreased risk of cardiovascular disease, including lowered blood pressure, improved plasma lipid profile, changes in coagulation and hemostatic factors, reduced incidence of obesity and type 2 diabetes mellitus, and improved metabolic control in individuals with established type 2 diabetes mellitus [2].

Rather than relying solely on body mass index (BMI), anthropometric variables, such as waist circumference (WC) and waist-to-height ratio (WtHR), are used to predict the risk of obesity-related diseases associated with intra-abdominal fat. As a result, the US Army has employed the WC formula to calculate the percent body fat (%BF), which is more closely related to intra-abdominal fat than to total body fat [3-5]. Studies indicate that high %BF and WC cutoff values are strongly associated with metabolic syndrome (MetS) risk, including atherosclerotic cardiovascular disease and type 2 diabetes mellitus [6-9]. As a result, the primary goal of military body composition regulations is to promote physical exercise and nutritional habits that ensure a physically capable military force ready to deploy at any moment [5].

In the military, the ideal fitness test is simple to administer, requires minimal equipment, and can be performed in the field [10]. The main components of a military fitness test are push-ups, sit-ups, and running tests, which are classified into muscular and aerobic endurance, respectively [11,12].

The Military Physical Training Manual of the Saudi Army consists of four physical fitness components: endurance, strength/power, mobility, and flexibility. Endurance is aerobic training based on the body's ability to intake and utilize oxygen (maximal oxygen consumption {VO_2_ max}), such as long-distance marching and jogging. Strength training is the maximal force produced by a muscle; for instance, core-strengthening exercises, such as the plank, are conducted to increase the load-carrying capacity of the abdomen and back muscles surrounding the spine. Mobility training aims to improve recruits' speed, jumping, and direction-changing abilities. Finally, flexibility training, such as static and dynamic stretching, is conducted to achieve or maintain an appropriate muscle length-tension relationship across the joints of recruits [11]. Several studies have demonstrated that these fitness components are essential for developing and sustaining physically balanced, injury-resistant, and mission-ready military members [13]. However, to the best of the researchers' knowledge, no research has been conducted on the impact of military programs on the physical fitness of new Saudi recruits. Such research could provide a foundation for future Saudi military studies on the effectiveness of military programs for the physical fitness of new recruits. This study aims to determine Saudi medical recruits' fitness outcomes and self-reported activity levels after a 10-week IMTC.

## Materials and methods

This experimental study comprised 104 male physicians aged 25-29 enrolled in a military freshman program for medical officers from December 13, 2020, to February 22, 2021, at the Prince Sultan Military College of Health Sciences. The participants were deemed fit based on an initial military health inspection that ensured they were free from any health condition, pharmacological treatment, or other interferences, such as smoking, which could affect the study results. Participation in this study was voluntary. Over 10 weeks, all participants were engaged in 25 physical training sessions, each lasting 60 minutes, as described in Table 1. The training sessions covered all the four components of physical fitness outlined in the military manual; see Table 2.

**Table 1:** Study protocol that includes physical training sessions.

Months	December			January		January		February		
Weeks (n)	1	2	3	1	2	3	4	1	2	3
Sessions (60 minutes each) (n)	3	3	3	3	3	2	2	2	2	2

**Table 2:** Study protocol that includes the load distribution during the sessions in each week (presented as percentage).

Physical component	December (three weeks)	January (weeks 1-2)	January (weeks 3-4)	February (three weeks)
Endurance	80.5%	80.5%	41.7%	41.7%
Strength/power	8.3%	13.9%	41.7%	41.7%
Mobility	8.3%	2.7%	8.3%	8.3%
Flexibility	2.7%	2.7%	8.3%	8.3%

The anthropometric variables and body compositions assessed were body weight (BW) (kg), height (cm), BMI (kg/m^2^), WC (cm), WtHR, estimated %BF that was computed using a published US Army quantitative equation [14-16], and estimated lean body mass (LBM; 0.407 × weight {kg} + 0.267 × height {cm} - 19.2) [17]. The fitness tests included a one-minute sit-up test (n), push-up test (n), Cooper's 12-minute run/walk test (m), and relative VO_2_ max (distance covered in meters - 504.9 ÷ (44.73 × 1 mL⸱kg^-1^⸱minute^-1^)) [17]. In addition, a seven-item version of the International Physical Activity Questionnaire (IPAQ) was used [18].

The IPAQ is a self-reported measure of physical activity that can be used to compare populational physical activity levels across countries. The questionnaire was used to collect information regarding the number of days and average time spent per day of being physically active. It was also used to record vigorous-intensity, moderate-intensity, and walking activities and sitting in the last seven days. The IPAQ was a subjective measure of the recruit's physical activity levels at the beginning and end of the study.

The study lasted 10 weeks, and the data were gathered during the second and 12th weeks of training. The sessions were conducted in the early morning during the winter season. The study was approved by the Institutional Review Board of Prince Sultan Military College of Health Sciences (IRB-2021-PT-003). This study was carried out following the tenets of the Declaration of Helsinki. Due to the coronavirus disease 2019 (COVID-19) pandemic and the importance of minimizing contact for safety, physical exercise was reduced from three sessions during the first five weeks to two sessions during the remaining five weeks.

Statistical analysis

We assessed data for normality using a Kolmogorov-Smirnov test. Data were reported as mean ± SD if normally distributed (parametric) and as median (interquartile range, IQR) if not normally distributed (non-parametric). The chi-squared or Fisher exact tests were used to compare categorical variables. For non-parametric paired data, the Wilcoxon signed-rank test was employed. The Statistical Package for Social Sciences (SPSS) software version 26 (IBM SPSS Statistics, Armonk, NY) was used to analyze our data.

## Results

A total of 104 subjects were analyzed and included in the primary analysis. The subjects had a mean ± SD age of 26.15 ± 1.07 years. The baseline weight (kg) was 79 kg (70.63-85.5), with a median BMI of 26.23 (24.23-29.03). The baseline %BF for the recruits was 20.06 (15.81-25.62), and the LBM was 59.11 kg (55.41-62.17); see Table 3.

**Table 3:** Demographic data of the subjects. IQR: interquartile range

Variable	Value (mean ± SD or median IQR or percentage)
Age	26.15 ± 1.07
Education (doctor of medicine)	104 (100%)
Marital status (married)	104 (100%)
Height (cm)	172 (169-175)
Weight (kg)	79 (70.63-85.5)

The anthropometric variables

We compared pre-course with post-course values of the anthropometric variables. There was a significant decrease in the BW (79 kg {70.63-85.5} versus 78 kg {71.05-84.95}) (P = 0.01), and this was associated with a reduction in the BMI (26.23 {24.23-29.03} versus 26.00 {24.12-28.44}) (P = 0.01) and %BF (20.06% {15.81-25.62} versus 19.62% {15.69-24.18}) (P = 0.002). The remaining variables were associated with a significant reduction (P < 0.05 for all) following the course, as seen in Table 4.

**Table 4:** Changes in anthropometric variables in new medical recruits from pre-training to post-training (median, IQR, or mean ± SD). BMI, body mass index; LBM, lean body mass; IQR, interquartile range

Variable	Pre-training	Post-training	P-value
Weight (kg)	79 (70.63-85.5)	78 (71.05-84.95)	0.01
BMI (kg/m^2^)	26.23 (24.23-29.03)	26.00 (24.12-28.44)	0.01
%BF	20.06 (15.81-25.62)	19.62 (15.69-24.18)	0.002
LBM (kg)	59.11 (55.41-62.17)	58.64 (55.19-61.73)	0.01
Waist circumference (cm)	88.90 (81.28-96.52)	86.00 (81.28-91.44)	0.005
Waist-to-height ratio	0.52 (0.47-0.56)	0.51 (0.48-0.54)	0.003

Physical fitness variables

There were significant changes in all the physical fitness variables. The push-up number has increased from 19.00 (14.25-25.00) to 30.00 (22.00-36.00) (P = 0.001), and this was associated with an increase in the one-minute sit-ups (from 30.00 {23.25-37.00} to 40.00 {32.00-47.00}) (P = 0.001) and 12-minute test (from 1767.50 m {1437.50-1979.00} to 1993.50 m {1804.25-2315.00}) (P = 0.001). Table 5 shows the other significant changes in the 12-minute test and the relative VO_2_ max pre- and post-IMTC.

**Table 5:** Changes in physical fitness variables in new medical recruits from pre-training to post-training (median, IQR, or n {%}). IQR, interquartile range; VO_2 _max, maximal oxygen consumption

Variable	Pre-training	Post-training	P-value
Push-ups (n)	19.00 (14.25-25.00)	30.00 (22.00-36.00)	<0.001
One-minute sit-ups (n)	30.00 (23.25-37.00)	40.00 (32.00-47.00)	<0.001
Twelve-minute test (m)	1767.50 (1437.50-1979.00)	1993.50 (1804.25-2315.00)	<0.001
Relative VO_2_ max (mL⸱kg^-1^⸱minute^-1^)	28.23 (20.85-32.96)	33.28 (29.05-40.47)	<0.001

Although all participants were invited to complete the IPAQ, only 83 volunteered. The comparison of pre-IPAQ with post-IPAQ demonstrated a percentage improvement in walking activity (15%-82%) and vigorous physical activity (17%-49%) after joining the IMTC, but this was not statistically significant.

## Discussion

This study aims to determine Saudi medical recruits' fitness outcomes and self-reported activity levels after a 10-week IMTC. The IMTC improved the new recruits' body composition, physical fitness, and self-reported physical activity levels. The Saudi Army's Military Physical Training Manual guides the fitness training of new recruits. However, to the researchers, this was the first study to determine the effects of Saudi Arabia's IMTC on body composition, physical fitness, and self-reported physical activity levels. The investigators observed a significant decrease in WC, which is consistent with previous studies in the literature [1,19]. WC is a crucial marker for identifying MetS risk, and the optimal cutoff values vary greatly depending on ethnicity [20,21].

The WC cutoff value for MetS risk in the Saudi population is 92 cm [22], which is higher than the post-training WC average of 86.36 cm in this study. Similarly, the %BF in this study (19.62%) was in the range of the US Army body fat percentage for males who are military service candidates (18%-26%) [23]. The favorable decreases in post-training WC, %BF, and WtHR emphasize the importance of incorporating a daily physical activity routine for this age group. In contrast, LBM decreased unfavorably, which could be attributed to the duration of the study. Prior literature has shown increased LBM in studies that lasted ≥12 weeks [1,19]. It should be noted that even though some studies [1,19] have comparable training components to our research, the percentage of endurance/aerobic training was practically half of ours (40% compared to 80%). This might have contributed to weight loss and LBM reduction in our study than theirs. In addition, a more significant percentage of IMTC in this study was endurance/aerobic training than resistance training, which may further explain the LBM decline [24,25].

All aspects of physical fitness improved after the IMTC. Although significant, the impact was less pronounced than that reported in the 12-week study conducted by Campos et al. in 2017. Training time and load may account for the difference in the results, as prior studies used a 48-hour training program [1], whereas this study only employed a 25-hour training program. Additionally, the study participants in Campos et al. [1] were aged 18-19 years, younger than those in this study, aged 25-29 years. This difference may have affected the anthropometric features and physical fitness. Despite the decreased training time and load during the last five weeks of the study, the self-reported IPAQ revealed a percentage improvement in walking activity (15%-82%) and vigorous activity (17%-49%) after joining the IMTC (P > 0.05 statistically not significant). The latter percentage increase suggests that higher levels of daily physical activity, such as heavy weight lifting and aerobics, as specified in the IPAQ, were embraced outside the military training program.

This study's findings indicate that physical training based on the Military Physical Training Manual of the Saudi Army promotes changes in morphological and physical fitness and self-reported physical activity level. The results provided preliminary evidence that the 10-week periodized physical training is a factor in the chronic adjustments of the military's body composition and physical fitness. In addition, it supports the notion that large-scale monitoring projects of military physical training can be conducted utilizing low-cost, simple protocols. Understanding how military training improves medical recruits' fitness implies that medical schools can adopt similar programs to enhance future healthcare professionals' health. Physical activity in medical school may boost physicians' health and resilience. The outcomes may impact military training. Studying how a 10-week training program affects fitness may help the military enhance medical recruits and other branch training regimens and preparedness. The study encourages more research into fitness programs, training intensities and durations, and the psychological benefits and downsides of military-style training for healthcare personnel. Future research should examine the training's long-term health effects and advantages beyond the 10 weeks.

This study had several limitations. One limitation is that the participants' caloric intake was not monitored throughout the 10-week study. Therefore, the reduction in LBM and %BF cannot be attributed solely to the physical training program. However, all participants enrolled in the IMTC followed the same food routine throughout the study. Furthermore, although the anthropometric and physical variables improved, the maximum fitness outcome was likely not achieved, as physical exercise sessions were reduced due to COVID-19 safety procedures. We lacked a control group and female representation, which would have strengthened our assessment. Finally, because the study cohort only included licensed physicians who joined the Saudi medical services, not younger combat soldiers, the study's findings cannot be generalized to all Saudi combat soldiers. Future studies should examine the impact of including a greater proportion of resistance training on lean body mass (LBM) during the IMTC and the impact of caloric intake on the body composition of military recruits.

## Conclusions

The study's findings, to the best of the researchers' knowledge, indicate that engaging in physical training according to the Saudi Army's Military Physical Training Manual can lead to positive improvements in body composition, physical fitness, and physical activity levels.
